# Application of Graphite Electrode Plasma Heating Technology in Continuous Casting

**DOI:** 10.3390/ma15072590

**Published:** 2022-04-01

**Authors:** Yong Wang, Jingxin Song, Nailiang Cheng, Zhenhe Guo, Jingshe Li, Shufeng Yang, Mengjing Zhao, Cun Wang

**Affiliations:** 1 School of Metallurgical and Ecological Engineering, University of Science and Technology Beijing, Beijing 100080, China; b20180094@xs.ustb.edu.cn (Y.W.); lijingshe@ustb.edu.cn (J.L.); b20180093@xs.ustb.edu.cn (M.Z.); a9800780@ustb.edu.cn (C.W.); 2 Baoteel Shanghai Meishan Iron and Steel Ltd., Nanjing 210039, China; 605833@baosteel.com (N.C.); 605834@baosteel.com (Z.G.); 3 Baosteel Central Research Institute Meishan R&D Center, Nanjing 210039, China

**Keywords:** plasma heating, continuous casting, graphite electrode, quality

## Abstract

In this study, the industrial, experimental effect of a plasma heating system in the form of graphite electrode in the tundish of double-strand slab caster was evaluated for the first time. The system uses three graphite electrodes, two of which are cathodes and one of which is an anode, to form a conductive loop through molten steel in the tundish. The system is built on an old two-strand slab caster and is installed on the premise that the original ladle tundish equipment remains unchanged. The normal working power of the system is up to 1500 kW, and the heating rate of molten steel in the tundish can reach 1.0 °C/min under conditions of 5 t/min total steel throughput and a tundish capacity of 50 t. After the system was put into operation, the purity of molten steel undergoing heating was investigated. The sample analysis of low carbon steel and ultra-low carbon steel before and after heating showed that the contents of N and O in the steel did not increase, while the size of the oxide inclusions near the heating point increased but showed little change in terms of the overall quantity. This process benefited from the addition of inert gas during the heating process to control the atmosphere in the heating area, which prevents reoxidation. The sample analysis also showed that there is no obvious carbon absorption phenomenon after heating, and the fluctuation in C content is within 0.0001%, which is consistent with the general production results. By using this system, the temperature of molten steel in the steelmaking process can be reduced by 10~15 °C, allowing continuous low superheat casting to be supported, which is helpful for reducing production costs and improving the solidified structure inside the slab. The results of the study show that the plasma heating technology can be applied to the continuous casting of low carbon–nitrogen steel slabs, which shows the benefits of reducing emissions and improving production efficiency.

## 1. Introduction

Molten steel temperature in tundish is the most important technical parameter in the continuous casting process. With the development of high-speed casting technology, it has been gradually realized that the influence of molten steel temperature in the tundish cannot be ignored. Reasonable temperature control cannot only improve productivity [1] and reduce the production cost but also improve the internal quality of continuous casting slabs [2,3,4,5,6]. Low temperature and constant temperature can stabilize the parameters such as superheat and casting speed, simplify the operation of the pouring process, and prevent steel leakage during continuous casting. Furthermore, the low superheat can refine grains, increase the proportion of equiaxed grains [7,8], and reduce the central segregation of the continuous casting slab [9,10,11,12,13].

Controlling the molten steel temperature in a tundish is one of the most important factors for improving productivity and solidification quality. The actual production shows that the large temperature drop of molten steel is caused by thermal radiation and heat absorption of refractory materials. In order to ensure smooth pouring, the high-temperature route is generally adopted [14,15,16]. From the tapping of the previous process to the completion of pouring, the temperature drop of molten steel is up to 55 °C, and the temperature change range of tundish during pouring is 20–30 °C. Tundish heating technology can flexibly compensate for the energy loss in the transfer and pouring process [17], reduce the tapping temperature in the previous process, and, more importantly, it can accurately control the superheat of tundish molten steel in the range of 10 °C.

Due to the characteristics of plasma, such as energy concentration, high temperature, easy control, fast heating response, and not introducing pollution to molten steel, plasma tundish heating (PTH) has attracted extensive attention in the 1980s and 1990s, and single or twin water-cooled metallic torch plasma heating equipment was developed. However, due to the short service life of electrodes, low heating efficiency, high operation cost, and heavy bottom electrode installed on tundish, plasma heating tundish technology has not been widely used [10,18,19,20,21,22,23,24]. In addition, the tundish induction heating equipment has a large volume, and the tundish capacity is 20%. It is also limited by the geometric shape of the tundish and refractory materials, and the maintenance cost is high. More importantly, at present, the heating effect of induction heating on large capacity tundish is limited [25]. In view of this, Beijing University of science and technology and Beijing Aobang Advanced Materials Co., Ltd., Beijing, China jointly designed and developed multi graphite electrode plasma heating equipment.

For the first time in China, the three-electrode DC plasma heating system was installed and applied in the 50 tons tundish of the two strands slab continuous casting machine. Based on its role in safeguarding the tundish plasma heating system, it can significantly reduce the outgoing steel refining temperature, which could reduce energy consumption and decrease carbon emissions. The application of this technology can also allow less low-temperature molten steel to be used and avoid broken pouring accidents. In addition, the stability of the casting speed and production rhythm is ensured, which facilitates the continuous and stable production of the whole process. In terms of product quality, the low-temperature casting method can reduce composition segregation in slabs and improve the uniformity of their solidification structure, thus improving the quality of the finished material. Therefore, tundish plasma heating technology has good application prospects for energy saving and improving production efficiency.

## 2. Plasma Heating Equipment and Layout

**Figure 1:**
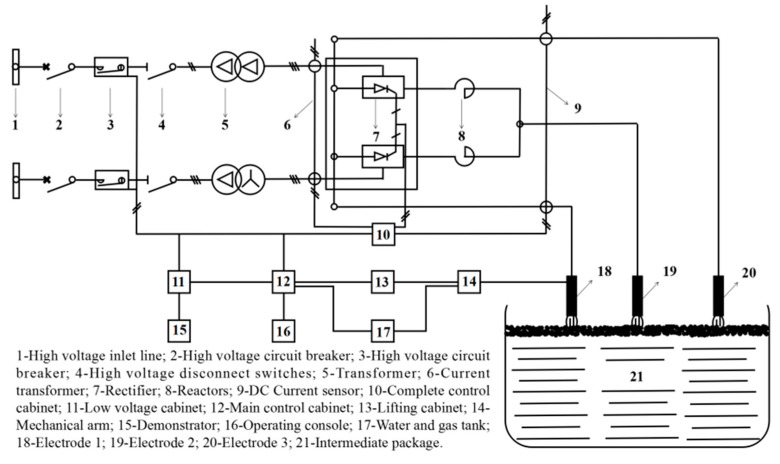
Schematic diagram of the tundish plasma heating equipment.

**Figure 2:**
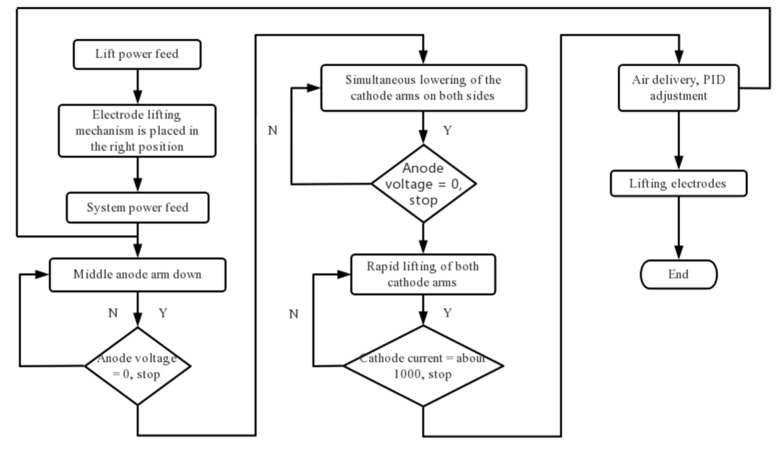
Program block diagram of tundish plasma heating system.

Water-cooling metallic torches have been used as tundish plasma heating equipment for a long time but have not been widely used due to various disadvantages. In recent years, new devices with graphite electrodes have been researched and applied [24,25]. The main equipment and heating procedures of the plasma heating system are shown in Figure 1 and Figure 2. Two 10 KV high-voltage power supplies are connected to the high-voltage distribution room to supply power to the tundish plasma transformer. A high-voltage contactor is used to remotely open and close the transformer. In order to facilitate maintenance, a high-voltage disconnector is set at the high-voltage side of the transformer, which is isolated and depressurized by the transformer and rectified by a rectifier; the smoothing is carried out through the smoothing reactor, and the DC power is transmitted to the graphite electrode of the plasma heating linear robot using a copper bar and water-cooled cable. Three plasma heating linear robots are arranged on the operation platform, and the operation room is equipped with an operation console and a lifting cabinet. The low-voltage cabinet provides low-voltage power for the PLC main control cabinet, operation console, lifting cabinet, and water–gas control box system. The position path of the electrode entering the tundish is taught through a teaching device. When the temperature of molten steel in the tundish is low, the plasma heating system is started using a key on the console. The plasma heating linear robot automatically enters the tundish according to the teaching position, starts the rectifier system, heats and starts the arc, and automatically maintains the set arc length according to the set parameters. When the molten steel in the tundish reaches the target temperature, one key stops, and the plasma heating linear robot automatically returns to the standby position.

**Figure 3:**
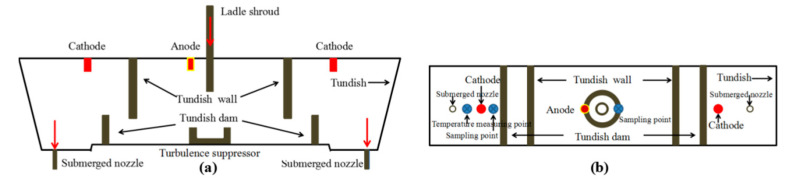
Layout diagram of equipment and test points: (**a**) front view; (**b**) vertical view.

In this project, this new DC plasma heating system with graphite electrodes was installed for the first time on a two strands slab casting tundish. The installation layout of tundish plasma equipment is shown in Figure 3; the device adopts three electrodes to form a current circuit through the liquid steel. The anode is in the middle, and the cathode is on both sides. Within the cathode are blowholes for argon, which is ionized at high temperatures to form a plasma arc. No special modifications of the caster or tundish are needed for this system.

## 3. Plasma Heating and Sampling Scheme

**Figure 4:**
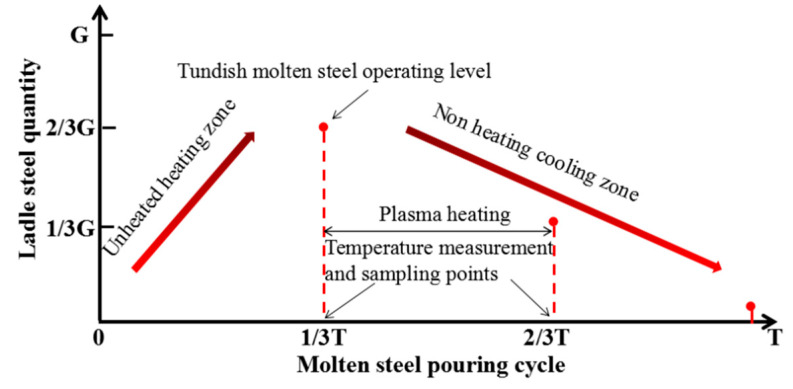
Schematic diagram of the plasma heating and sampling scheme.

As shown in Figure 4, the slab size is 1320 mm × 230 mm, the tundish working weight is 50 tons, and the caster throughput is 5 t/min. In the single ladle pouring cycle, due to the high temperature of the molten steel injected from the ladle, the molten steel temperature in the tundish initially increases in the early stage (ladle steel water quality 2/3 G), and the molten steel temperature then gradually decreases with pouring. Therefore, in order to ensure the stability of pouring temperature, 2/3–1/3 G of ladle steel water quality is designed for plasma heating. In order to detect the influence of plasma heating equipment on the pouring process temperature and liquid steel quality, continuous temperature measurement and sampling are carried out near the anode and cathode before and after heating to analyze the changes in nitrogen and carbon content and inclusion characteristics in the steel. The time and location of temperature measurement and sampling are shown in Figure 3 and Figure 4.

## 4. Application Results

The new device was applied in an actual casting process. The results are discussed below.

### 4.1. Effect on Tundish Temperature Increasing

**Figure 5:**
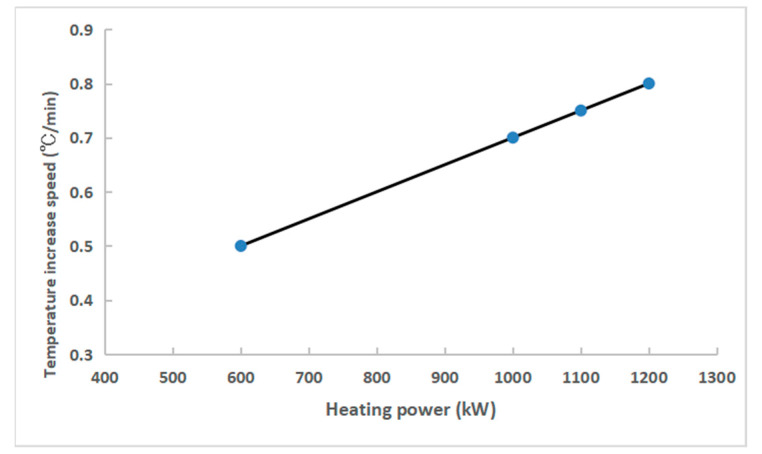
Temperature increase rate (°C/min) of liquid steel vs. heating power (kW).

To evaluate the temperature change based on power, the temperature of liquid steel in the tundish was continuously measured. When compared with traditional tundish heating equipment, the hollow graphite electrode is not easily damaged, has a long service life, and has a low maintenance cost. The three-electrode self-contained loop does not require adding a bottom electrode and additional tundish modification at the bottom of the tundish. The heating efficiency is high, the reaction speed is fast, and the heat loss of the molten steel in the tundish can be compensated in time and effectively [18,19,20,21,22,23,24,25]. The results show that there is a positive linear correlation between the heating rate and the heating power, as shown in Figure 5. Generally, the plasma heating condition is 50 t tundish with 5 t/min of steel passing through, and the heating power is 1500 kW. According to the relationship between the heating rate and the heating power, the heating rate of the molten steel at the outlet of the tundish is close to 1.0 °C/min.

**Figure 6:**
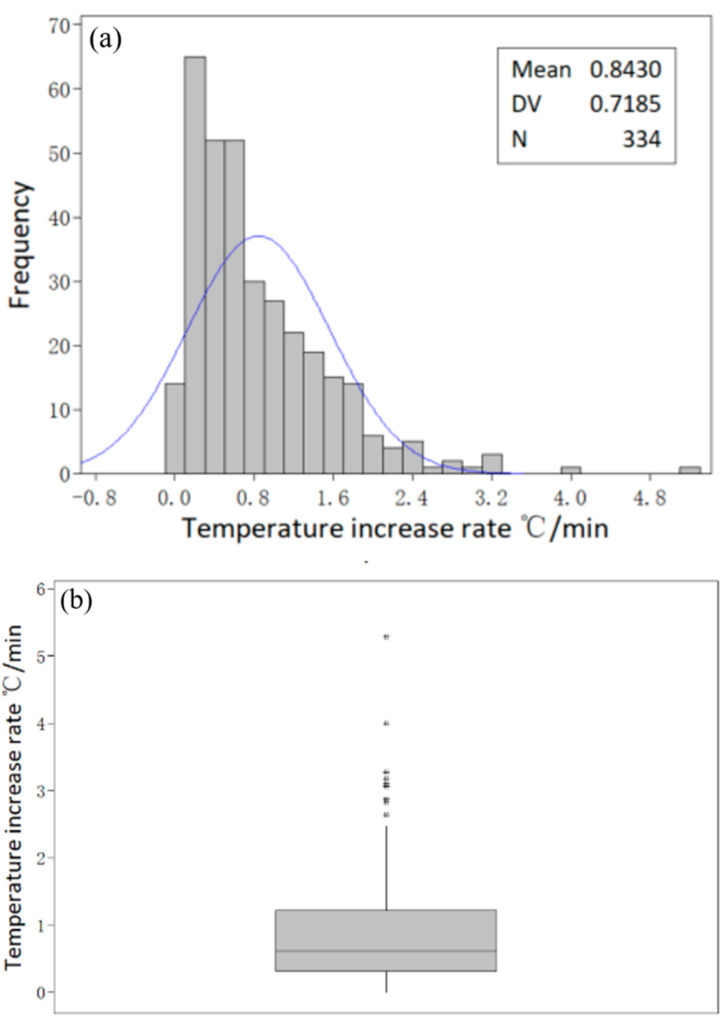
Temperature increase rate distribution at maximum heating power: (**a**) temperature rate histogram; (**b**) temperature rate boxplot.

The temperature data comes from the continuous temperature measuring instrument installed in the tundish. The following results are obtained from mass production data statistics: (1) the mean heating time is 9.2 min, and the maximum heating time is 25 min; (2) the mean temperature increase is 6.3 °C, and the maximum temperature increase is 24 °C; (3) the mean temperature increase rate is 0.8 °C/min, and the maximum temperature increase rate is 5.3 °C/min. The distribution of statistical data is shown in Figure 6.

There are large fluctuations in the temperature increase rate, and even zero or negative values. Through the in-depth analysis of the original date, it can be found that the temperature increase rate is low in the latter half of ladle pouring. The temperature of the molten steel itself decreased during this period, and the temperature drop was very significant. When heating and cooling balance out, the overall temperature performance is unchanged or slightly lower. In addition, the temperature increase rate in the first half of ladle pouring is faster, which is due to the temperature increase in the pouring process itself, which makes the temperature rise rapidly. In general, the average temperature rise rate reflects the heating capacity of the equipment. Overall, the average value of the temperature increase rate reflects the heating capacity of the device.

### 4.2. Effect on Tundish Temperature Distribution

A large number of tundish temperature data under different process conditions are counted, and the influence of tundish plasma heating technology on tundish temperature distribution is analyzed. Here, temperature refers to the statistics of all casting ladles, and temperature fluctuation refers to the difference between different casting ladles.

Under conditions where there is no tundish heating, the tundish superheat distribution of all ladles abides by the normal law Equation (1).

ΔT_TD_ ~ N (μ, σ^2^), (1)

**Figure 7:**
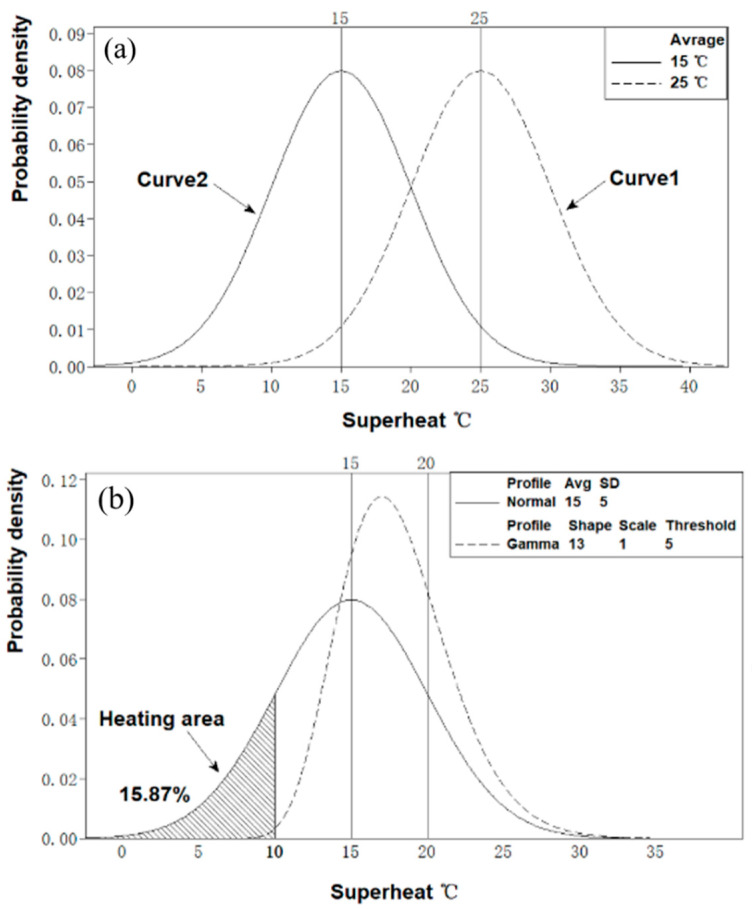
Analysis of tundish superheat distribution: (**a**) without heating; (**b**) with heating.

The superheat distribution is shown in Figure 7a. In general, the standard deviation σ is 5 °C according to the actual situation. For curve 1, the average value μ is 25 °C, and −3σ is 10 °C. For curve 2, the average value μ is 15 °C, and −3σ is 0 °C. The two curves represent different working conditions. Be aware that curve 2 represents the low limit superheat control procedure.

In the working condition, with tundish heating, the device can be set to start when the superheat of liquid steel falls below a certain standard value. As a result, the temperature fluctuation range in the tundish of different ladles can be reduced. For instance, the superheat distribution curve of ΔT_TD_ ~ N (15, 52) is shown as solid lines in Figure 7b. If heating starts when the superheat is below 10 °C, the lower limit of the range is increased to 10 °C, and the fluctuation range is reduced from 30 °C to 20 °C, which is shown as dotted lines in Figure 7b. The percentage of heating for the ladle is 15.87%. The new temperature distribution curve (dotted lines) abides by the gamma law, or positive skewness distribution Equations (2) and (3).

(2) $$f\left(x|a,b\right)=\frac{1}{b^{a}\Gamma \left(a\right)}x^{a-1}e^{-\frac{x}{b}},x>0$$

(3) $$\Gamma \left(a\right)=\int_{0}^{+\infty }x^{a-1}e^{-x}dx,a>0$$

**Figure 8:**
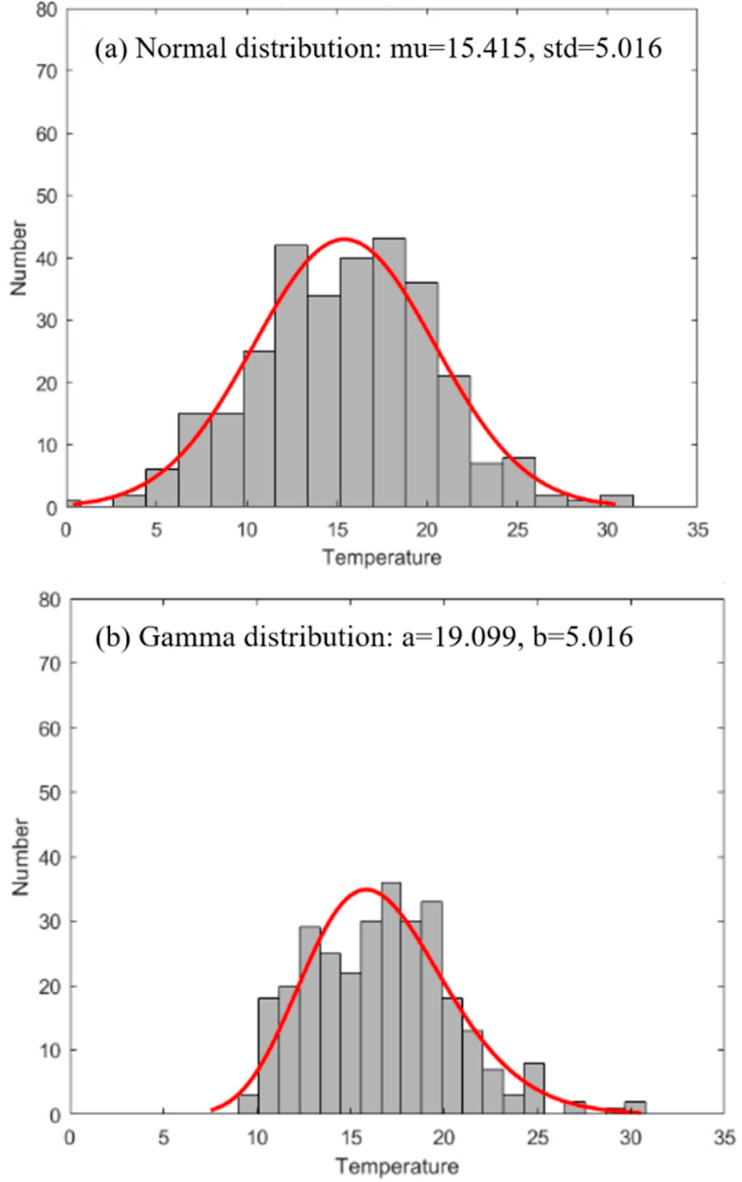

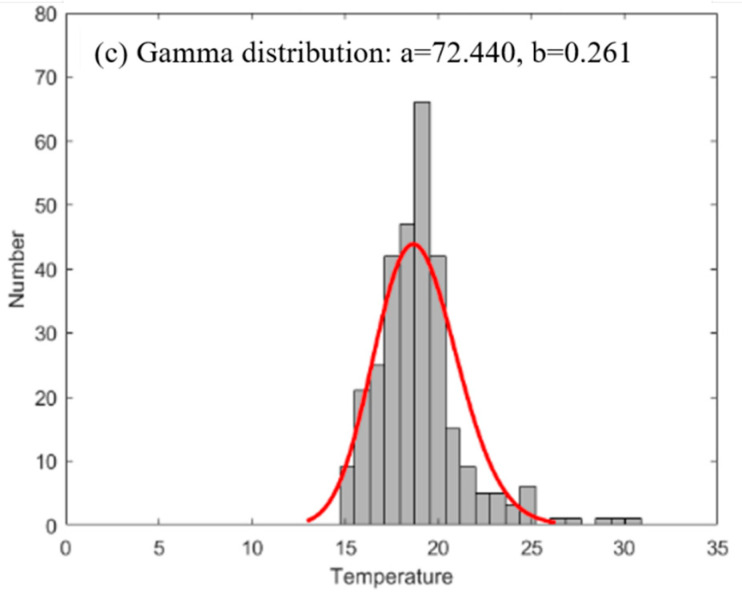
Distribution of tundish superheat for procedures (**a**) without heating; (**b**,**c**) with heating.

For the actual tundish temperature statistics, Figure 8 shows the superheat distribution of the tundish for different procedures.

Figure 8a, without heating, shows a normal distribution. Figure 8b is with heating, and heating starts when the superheat is lower than 10 °C. Figure 8c is also with heating, and heating starts when the superheat is lower than 15 °C. With the increase in the initial heating temperature, the temperature distribution range decreases as expected. The fluctuation range of superheat in the tundish can be greatly reduced by heating liquid steel, which is beneficial in terms of the stability of the production process and quality stabilization.

### 4.3. Effect on Temperature of Preceding Process

As the molten steel heat loss can now be compensated for in the tundish, both the temperature of the preceding process and superheat temperature can be reduced to save energy and reduce emissions. For this reason, a comparative test to decrease the tapping temperature of the preprocessing was carried out. Statistical test data are shown in Table 1. The average superheat of the refined station outlet during the test was 7 °C lower than usual. Still, the coefficient of variation (CV) was basically the same, and the dispersion degree of superheat was similar.

### 4.4. Effect on Carbon and Nitrogen Content of Steel

There is concern regarding the carbon element of the graphite electrode used for heating, specifically whether it results in carbon increases when entering the liquid steel, and nitrogen increase is also a potential issue. Therefore, the changes in carbon and nitrogen elements in ultra-low carbon steel slabs before and after heating were investigated. The results are shown in Table 2. It can be seen that the maximum difference in the carbon content of the slab before and after heating was 15.6%, which is consistent with the level of change for this kind of steel under a non-heating process. At the same time, due to the use of argon protection, the nitrogen content of the slab after heating was reduced by 6.2%. The results show that, compared with induction heating, although the graphite electrode plasma heating will introduce a certain amount of carbon and nitrogen, the protective atmosphere provided by the hollow argon blowing and the electrode control system can make the increase in carbon and nitrogen in the molten steel control extremely. The low range has no significant effect on the overall composition of the molten steel [18].

### 4.5. Effect on Inclusions in Steel

**Figure 9:**
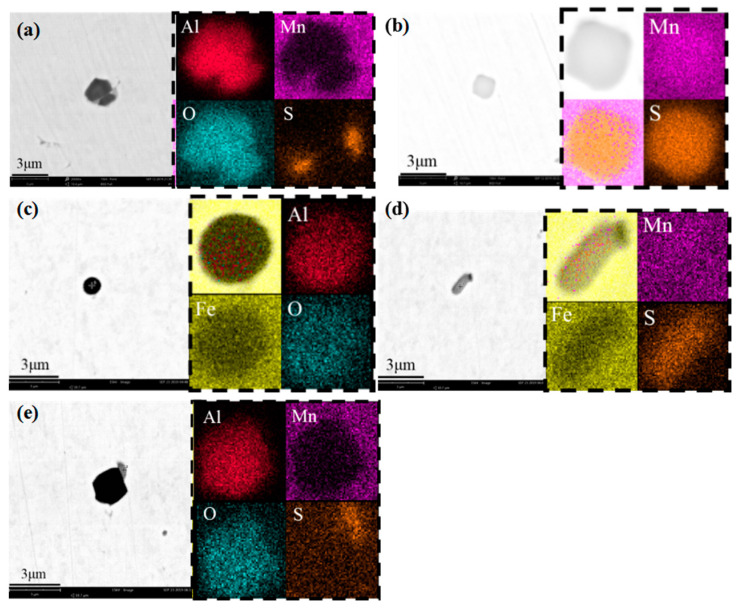
Comparison of inclusions in slabs before heating: (**a**,**b**) middle specimen; (**c**–**e**) edge specimen.

**Figure 10:**
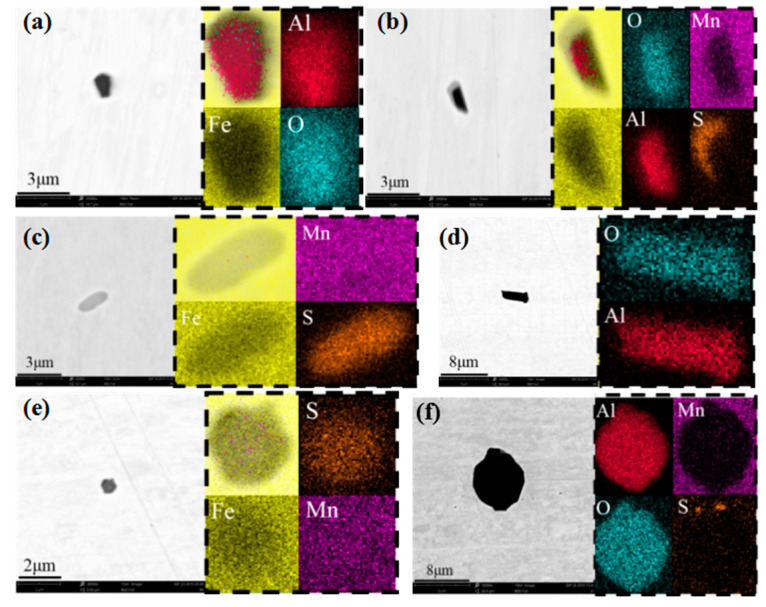
Comparison of inclusions in slabs after heating: (**a**–**c**) middle specimen; (**d**–**f**) edge specimen.

The low-carbon steel slabs were sampled before and after heating. The processed metallographic samples were observed by scanning electron microscopy (SEM); the inclusions were photographed, and the elemental composition was analyzed by an energy dispersive spectrometer. As illustrated in Figure 9, before heating, the typical inclusions in the steel were mainly the micro inclusions of deoxidation products MnS, Al_2_O_3,_ and composite Al_2_O_3_–MnS. No large inclusions formed by slag entrapment were found. In addition, the inclusions in the tundish were evenly distributed, and the size of MnS and Al_2_O_3_ inclusions in the edge (Figure 9c–e) and middle (Figure 9a,b) were mainly 1–3 μm. The size of the Al_2_O_3_–MnS composite was 2–3 μm. The typical inclusion types in the steel after heating were basically the same as those before heating, as shown in Figure 10. They were mainly the micro inclusions of MnS, Al_2_O_3,_ and composite Al_2_O_3_–MnS formed by deoxidation products, and no large inclusions were found. Different from that before heating, the inclusion size near the edge (Figure 10d–f) heating hot spot increased significantly, and the size of Al_2_O_3_ was 1–4 μm. The size of Al_2_O_3_–MnS composites was increased to 1–8 μm. The size of inclusions in the middle (Figure 10a–c) was basically the same as that before heating. The result shows that compared with induction heating, in the hot spot of plasma heating, the impact of plasma flow would lead to a certain level fluctuation. Still, the fluctuation degree was small, which would not lead to a large amount of slag in liquid steel. In addition, the molten steel at the impact point was exposed. Although there was an argon protective atmosphere, there was still slight local secondary oxidation. The high temperature destroyed the physical and chemical properties of the covering agent, reduced the adsorption capacity of inclusions, resulting in the increase of inclusion size at the heating point. Still, its influence range was small and had little impact on the overall quality of molten steel.

## 5. Conclusions

(1) Tundish heating technology has achieved a new milestone. Here, the first set of multi-graphite electrode DC plasma heating systems based on a two-strand slab caster was developed for the first time and realized in industrial applications. When compared with the traditional tundish heating system, the three-electrode self-contained loop does not require additional modifications to the tundish.
(2) The multi-graphite plasma heating system has a fast response speed and can timely supplement the temperature loss of the molten steel in the tundish in the later stage of pouring. When the outlet temperature of liquid steel at the refined stage is reduced by 7 °C on average, the tundish heating rate only needs to be 15%; the average heating time is 9 min/heat, the average temperature increase is 6.3 °C, and the average temperature rise increase is 0.8 °C/min.
(3) The analysis of the nitrogen and carbon content of steel before and after heating can determine the harmful effects of plasma heating technology on steel composition. When compared with induction heating, although in the hot spot of plasma heating, the inclusion removal ability of mold flux decreases, and the size of oxide inclusions increases. However, its influence area was small and had little impact on the overall quality of liquid steel.
(4) With the capability for tundish energy compensation, the temperature of liquid steel in the preceding process can be reduced as a whole, so the overall benefits are significant.

## Figures and Tables

**Table 1:** Statistics of outlet superheat of refined molten steel.

Condition	N Total	Mean	SD	CV
With superheat decrease	322	61.44	7.62	12.4
Without superheat decrease	1243	67.69	7.60	11.23

**Table 2:** Key element analysis (mass fraction).

Sample No.	Carbon Analysis (Mass%)			Nitrogen Analysis (Mass%)		
	Before Heating	After Heating	Difference	Before Heating	After Heating	Difference
1	0.00570	0.00580	1.8	0.0024	0.0023	−4.2
2	0.00600	0.00670	11.7	0.0024	0.0023	−4.2
3	0.00385	0.00445	15.6	0.00241	0.00239	−0.8
4	0.00370	0.00421	13.8	0.0023	0.00215	−6.2
5	0.00423	0.00383	−9.5	0.0023	0.00219	−4.8

## Data Availability

Not applicable.

## References

[1] Liang W., Mustoe T.N. (1998). Low superheat casting through control of tundish steel temperature. Steel Times.

[2] Sahai Y. (2016). Tundish technology for casting clean steel: A review. Metall. Mater. Trans. B.

[3] Dong Z.Y., Chen Q.Q., Yang Y.G., Shi B. (2013). Experimental and Numerical Study of Hydrodynamic Cavitation of Orifice Plates with Multiple Triangular Holes. Appl. Mech. Mater..

[4] Filippov G.A., Tyuftyaev A.S., Gadzhiev M.K., Yusupov D.I., Sargsyan M.A. (2016). Effect of stabilizing steel temperature in a continuous-caster tundish by the plasma method on the uniformity of the mechanical properties of plates after rolling. Metallurgist.

[5] Kittaka S., Wakida S., Sato T., Miyashita M. (2005). Twin-torch type tundish plasma heater “NS-plasma II” for continuous caster. Nippon Steel Tech. Rep..

[6] Ludlow V., Normanton A., Anderson A., Thiele M., Ciriza J., Laraudogoitia J., Van Der Knoop W. (2005). Strategy to minimise central segregation in high carbon steel grades during billet casting. Ironmak. Steelmak..

[7] Hui Y.J., Yu Y., Wang L., Wang C., Li W.Y., Chen B. (2016). Strain-induced precipitation in Ti micro-alloyed interstitial-free steel. J. Iron Steel Res. Int..

[8] Park J.Y., Oh K.H., Ra H.Y. (2001). The effects of superheating on texture and microstructure of Fe–4.5 wt% Si steel strip by twin-roll strip casting. ISIJ Int..

[9] Haque M.M., Ismail A.F. (2005). Effect of superheating temperatures on microstructure and properties of strontium modified aluminium–silicon eutectic alloy. J. Mater. Processing Technol..

[10] Pak Y.A., Filippov G.A., Yusupov D.I., Tyuftyaev A.S., Isakaev M.E., Sarychev B.A. (2014). Two-strand tundish with chambers for plasma heating of liquid metal. Metallurgist.

[11] Isakaev E.K., Tyuftyaev A.S., Mordynskii V.B., Filippov G.A., Pak Y.A., Yusupov D.I. (2014). Influence of steel temperature in continuous casting on its structure and properties. Steel Transl..

[12] Ferreira A.F., Chrisóstimo W.B., Sales R.C., Garção W.J.L., de Paula Sousa N. (2019). Effect of pouring temperature on microstructure and microsegregation of as-cast aluminum alloy. Int. J. Adv. Manuf. Technol..

[13] Ganguly S. (2007). Morphology and segregation in continuously cast high carbon steel billets. ISIJ Int..

[14] Bebber H.J. (1990). Casting temperature control using a plasma tundish heater. Steel Times.

[15] Moore C., Heanley C.P., Cowx P.M. (1989). Plasma tundish heating as an integral part of continuous casting. Steel Times Int..

[16] Wang C., Pan G., Yang C., Zhang J., Zhao P., Page A. (1997). Experimental investigation on plasma tundish heating. Iron Steel.

[17] Okorokov G.N., Donets A.I., Shevtsov A.Z., Sinel’nikov V.A., Yugov P.I., Zin’ko B.F., Krutyanskii M.M., Popov A.M. (1998). A heating tundish—The final link in a continuous steelmaking technology. Metallurgist.

[18] Fujimoto H., Tokunaga H., Iritani H. (1994). A High-Powered A.C. Plasma Torch for the Arc Heating of Molten Steel in the Tundish. Plasma Chem. Plasma Process..

[19] Tian J.Y., Zhang X.L., Li J.S., Wang X.Z., Wang C. (2017). Review of Plasma Heating Technology for Continuous Casting Tundish. Wide Heavy Plate.

[20] Barron-Meza M.A., Barreto-Sandoval JD J., Morales R.D. (2000). Physical and Mathematical Models of Steel Flow and Heat Transfer in a Tundish Heated by Plasma. Metall. Mater. Trans. B.

[21] Sha J., Qian H., Zhu M. (2000). Study on Distributions of Fluid Temperature in Tundish with Plasma Heating Using Water Modeling. Gold J..

[22] Wang Y., Zhao M.J., Yang S.F., Li J.S., Zhang G.X., Xi X.J. (2000). Physical simulation of tundish heated by plasma. Chin. J. Eng..

[23] Badie J.M., Bertrand P., Flamant G. (2001). Temperature Distribution in a Pilot Plasma Tundish: Comparison Between Plasma Torch and Graphite Electrode Systems. Plasma Chem. Plasma Process..

[24] Liu T., Zhao M., Yang S., Li J., Chen Y., Wang C. (2000). Industrial practice of tundish plasma heating. China Metall..

[25] Tang H., Wang K., Li X., Liu J., Zhang J. (2021). Improved metallurgical effect of tundish through a novel induction heating channel for multistrand casting. Metals.

